# Introduction to the Special Issue on “New Trends towards Automatic Vehicle Control and Perception Systems”

**DOI:** 10.3390/s130505712

**Published:** 2013-05-02

**Authors:** Vicente Milanés, Luis M. Bergasa

**Affiliations:** 1 California PATH Program, University of California, Berkeley, CA 94804, USA; 2 Department of Electronics, Polytechnic School, University Campus, 28805 Alcalá de Henares, Madrid, Spain; E-Mail: luism.bergasa@uah.es

Intelligent and automatic systems are making our daily life easier. They are able to automate tasks that, up to now, were performed by humans, freeing them from these tedious tasks. They are mainly based on the classical robotic architectures where the stages of perception—using different sensor sources or even a fusion of a set of them—and planning—where intelligent control systems are applied—play a key role. Among all of the fields in which intelligent systems can be applied, transport systems are considered one of the most promising ones since over one million fatalities—including drivers, pedestrians, cyclists and motorcyclists—are registered each year worldwide and they can definitively help to reduce these figures.

The growth in the number of drivers in the last decades, and consequently in the number of vehicles, has made traffic accidents become a major concern in the road transportation sector. Solutions such as building new transport infrastructures—specifically in urban areas where space limitations make it almost impossible—or increasing fines for inappropriate driving have not achieved the goal of reducing the number of fatalities, either in urban environments or highways. New policies on the part of the Governments are focused on the development of a safer, more reliable and more efficient transportation supported by intelligent systems.

The field of research in which all these topics fall into is well-known as intelligent transportation systems (ITS). ITS focuses on improving road safety by acting on two possible aspects: on the one hand, the infrastructure and the possibilities of optimizing traffic flow, and on the other hand, acting on the cars with the development of Advanced Driver Assistance Systems (ADAS) and the possibilities of optimizing traffic safety. The final goal is trying to reduce the number of collisions, or at least to mitigate their consequences. In this connection, one of the currently hot topics in the road transportation field is the development of intelligent devices and systems for improving both traffic flow and safety. New advances in positioning, navigation or perception systems can clearly contribute to these goals. Additionally, these systems can also be adapted for the development of service robots in an effort to increase the quality of life for metropolitan area citizens.

Bearing the goal of helping people in their daily life in mind, sensors are critical since they can be used to detect potential risk situations in advance. In the automotive industry, size and cost are two fundamental limitations when it comes to introduce new ADAS. Recently, intelligent functionalities have been included in mass-produced vehicles, such as automatic parking systems—mainly based on ultrasonic sensors—where steering wheel, brake and throttle pedal are automatically managed to park the car without driver intervention; blind spot detection—mainly based on vision sensors—where a visual or audible signal indicates a potential risk situation for lane-change maneuvers because a vehicle is driving pretty close in an adjacent lane; or the adaptive cruise control system—mainly based on lidar/radar technology—where longitudinal vehicle control is automatically regulated to adapt its speed to the preceding car in a safe and reliable manner.

In spite of the significant contributions of all of these advances, there is still a long way to go. More sophisticated systems will have to be developed towards the up-to-now utopia of driverless cars driving in real traffic conditions. To this end, the combination of intelligent autonomous systems capable of analyzing all traffic conditions and sensors for detecting all potential situations in which vehicles are involved, will definitively contribute to improve road transportation.

As it was previously stated, perception is the first stage in the classical robotic control architecture and, consequently, the initial critical task when it comes to manage robot behavior because it gives information about its environment and the robot itself. Advances in this field will lead to the development of faster, smaller, more autonomous and more accurate robots. Concerning to this topic, this Special Issue presents some perception systems based on the fusion of different sensors such as: monocular and stereo vision, laser, sonar, GPS, inertial sensors, radar, *etc.*, applied to the localization, navigation, mapping and interaction of different prototypes. Important sensorial research for indoor ground, all-terrain rovers, humanoids, underwater and aerial robots, or even for assisting the humans, is being presented in the papers.

For the planning stage in the robotic control architecture, a proper management of the received information coming from the perception phase—*i.e.*, sensorial systems—has to be done. Control systems use the most valuable information from the perception stage in order to develop intelligent behaviors for the robotic systems. Concerning to this topic, this Special Issue presents some automatic control systems based on robust control theory, artificial intelligent techniques or advanced filtering systems for obtaining a good robot behavior or considerably improving its position and navigation strategies. The significant contribution of experimental papers for the Special Issue where the systems are not only tested in simulations environments but also in real experimental platforms where more challenging and unexpected situations occur is also noteworthy.

This Special Issue traces in part its origin to two workshops—the Workshop on Perception in Robotics and the Workshop on Navigation, Perception, Accurate Positioning and Mapping for Intelligent Vehicles—organized during the IEEE Intelligent Vehicles Symposium held in June 2012 in Alcalá de Henares (Spain). The workshop proceedings were successful, and a Special Section of the Open Access Sensors journal dealing with the same theme was planned. A call for papers was issued. The received papers accepted after a rigorous review and revision cycle are included in this Special Section. The thirty three papers that appear in this Special Issue cover the full range of new trends in perception and autonomous control applied to Intelligent Transportation Systems, specially focused in Intelligent Vehicles and Robotics. These papers are summarized as follows.

## 1. Papers in the Special Issue

Papers presented in this Special Issue can be divided in three main groups according to the device where the control or the perception systems is applied: humans, robots and intelligent vehicles.

### 1.1. Contributions Related to Humans

In [1] an algorithm for estimating a pedestrian location in an urban environment is presented. The algorithm is based on the particle filter and uses different data sources: a GPS receiver, inertial sensors, probability maps and a stereo camera. Authors claim that the algorithm is able to estimate a pedestrian's location with an error smaller than 2 m 90% of the time.

The aim of [2] is focused on the design of an obstacle detection system for assisting visually impaired people. A stereo camera carried by the user is employed as sensor and a dense disparity map is computed from the images. The system is completed with acoustic feedback. The system is validated by means of real tests using four volunteers.

### 1.2. Contributions Related to Robots

In [3] a matrix Kalman filter (MKF) using as inputs several on-board sensors has been implemented for an integrated indoor navigation system based on a 4 wheels robotic platform. The MKF rearranges the original nonlinear process model in a pseudo-linear process model. They employ the Lie derivatives criterion about observability rank to verify the conditions under which the nonlinear system is observable.

A modified-FastSLAM algorithm is proposed in [4] for the navigation of an open-frame autonomous underwater vehicle (AUV) using as active sensor a mechanical scanning imaging sonar. Experimental results show the authors' proposal is much more accurate and effective compared with the methods of the current state-of-the-art.

In [5] the problem of autonomous exploration of unknown environments with single and multiple robots is analyzed. Its authors propose an approach based on the combination of a behavior-based navigation and an efficient data structure where previously visited regions are stored. Experiments performed using a robotic simulator and a real platform show a good behavior from the proposed system.

In [6] the authors present an adaptive fusion method to improve the accuracy and reliability of the altitude measure for small Unmanned Aerial Rotorcraft (UAR) during the landing process. The effectiveness of the proposed method is proved by static tests, autonomous landing and hovering flight tests.

In [7] an outdoors laser-based pedestrian tracking system using a set of mobile robots is presented, where each of them operates close to the others. Each robot detects pedestrians from its own laser scan image, tracks them and broadcasts the tracking information to multiple robots. Using this cooperative strategy, all the robots have the information any of them detects. The simulation and experimental results show that this proposal works better than conventional individual tracking does.

The work in [8] shows a 3D terrain reconstruction technique from a mobile robot. Authors propose a new ground segmentation method for a voxel map. Authors show the time required for ground segmentation is faster than for data sensing and it can be applied in real-time.

Work developed in [9] outlines a cross-coupled controller for a 4-wheel-robot, which optimizes the wheel motors' control algorithm to reduce slip effects that could appear with conventional controllers. Experimental results were carried out using an all-terrain rover working in an agricultural terrain. They show the effectiveness of the proposal when it comes to reduce slippage and posture errors in the vehicle.

In [10] a new scheme for Doppler Velocity Log (DVL) aided Strapdown Inertial Navigation Systems (SINS) alignment using Unscented Kalman Filter (UKF) for AUV is presented, which allows large initial misalignments. Experimental results show that the proposed DVL-aided alignment model is works independently of any initial heading errors.

The work in [11] presents a system composed by a ground and an aerial robot that cooperate sharing sensor information. The terrestrial robot is able to navigate in an unknown large environment aided by visual feedback from the aerial robot through an on board camera. The proposal shows outstanding results in SLAM applications in large outdoor environments.

Authors in [12] implement a dynamic visual memory to store the information gathered from a moving camera on board a robot. An attention system to choose where to look with this camera and a visual localization algorithm based on this visual memory complete the authors' proposal. Experimental results, both with simulated and real Pioneer and Nao robots, validate the proposal in office scenarios.

The work developed in [13] implements a low-cost tele-operated system in order to replicate the movements in a small humanoid robot. A feedback stability control and a fuzzy control based on low-cost open platforms have been developed. Some experimental results validate the system.

The goal of [14] is to solve the problem of dynamic obstacle avoidance for a mobile platform by using the stochastic optimal control in terms of safety and energy efficiency framework to compute paths. Authors propose a 3D extension of the Bayesian Occupancy Filter (BOF) to deal with the noise in the sensor data. Some experimental results highlight the advantages of the proposal against classical algorithms.

The work in [15] is concerned with the use of a mobile ground-based panoramic radar sensor. This is able to deliver both distance and velocity of multiple targets in its surrounding. The authors' purpose is to study data distortion and Doppler effect in order to estimate vehicle's movements. Some radar-only localization and mapping experimental results are presented for a ground-vehicle moving at high speed.

### 1.3. Contributions Related to Intelligent Vehicles

In [16] a dedicated public urban transportation service access system named Mobi+ has been introduced. This facilitates the mobility of disabled, wheelchair and blind passengers. So far, the Mobi+ system has been implemented on the buses and stations of line ‘2’ in the city of Clermont-Ferrand (France). The experimental results show that Mobi+ provides an effective urban bus access service for people with disabilities and it is easily to deploy in the buses and at bus stations.

In [17] a real time speed supervisor based on road sign recognition able to work in urban and non-urban environments is presented. The system can recognize 135 road signs and sends warning messages to the driver. The advantages and disadvantages of the two main methods traditionally used for detection and recognition of road signs [template matching (TM) and neural networks (NN)] are shown.

The authors of [18] describe the perception system designed for the intelligent vehicle that won the 2010 Future Challenge (SmartV-II). This system uses the fusion of multiple lasers and cameras to realize several functions of autonomous navigation (road curb detection, lane detection and traffic sign recognition). The experimental results validate the proposed system.

A reliable freestanding position-based routing algorithm (FPBR) for highway scenarios in the context of Vehicular *Ad Hoc* Networks (VANETs) is proposed in [19]. FPBR performance is compared to one of the leading protocols for highway scenarios showing that FPBR yields similar results, when considering free space propagation conditions, and outperforms the leading protocol when considering a realistic highway path loss model.

The work in [20] presents a two-layer based enhanced map for supporting navigation in urban environments. One layer is dedicated to describe the drivable road focusing on the accurate description of its bounds. The other layer depicts building heights and locations enabling the detection of non-line-of-sight signals coming from GPS in an indirect view. The methodology for creating these enhanced maps is shown in the paper.

The study in [21] presents the development of an intelligent parking service called iParking. With this service users, parking facilities and service providers are connected through Internet. The client software is an application running on a smartphone based on a precise indoor positioning solution. Experimental results show the iParking working in a real parking environment at a shopping mall.

In [22] authors describe a framework to combine experts' judgments for the prevention of driving risks in a cabin truck simulator. Three experts were asked to evaluate the driving risk using a Visual Analog Scale. Numerical results show that the proposal is suitable for embedding in real-time systems.

The paper in [23] presents a vehicle dynamics prediction system consisting of a sensor fusion system and a vehicle identification one. Comparing with most important works in this field, the proposal improves the prediction accuracy both by incorporating more vehicle dynamics to the prediction and by minimizing the vehicle modeling errors.

The work in [24] presents an integrated approach to examine the force exertion and movement pattern during continuous steering movement (CSM) of the upper extremity (UE). These findings help us to understand CSM and have important implications for practice in clinical training.

In [25] the authors present a forward collision warning system, based on a laser scanner, which is able to detect several potential danger situations. Decision algorithms determine the most convenient manoeuvre, act on the actuators of the ego-vehicle and transmit this information to other vehicles using V2V communications. The system has been tested for overtaking manoeuvres under different real scenarios.

Authors in [26] detail an advanced GNSS/IMU fusion system based on a context-aided Unscented Kalman filter for navigation of Intelligent Vehicles in urban conditions. An exhaustive analysis has been carried out with available data to show the effectiveness of the proposal.

The work in [27] proposes a method for monitoring driver safety levels using a data fusion approach and developed in the form of an application for an Android-based Smartphone device. Experimental results in simulation show the benefits of the fusion in providing a more effective driver safety monitoring.

Using a 3D urban model to forecast satellite visibility in urban contexts to improve GPS positioning is the main topic of the paper presented in [28]. The authors propose a virtual image processing that detects and eliminates possible faulty measurements. This closed-loop real-time proposal has shown very promising test results.

In [29] authors test a system that uses low-cost sensors, based on MEMS technology, coupled with information derived from a video camera on-board a two-wheel motor vehicle. They present a method for the reconstruction of the trajectory of a “Vespa” scooter as alternative to the well-know approach based on GPS/INS fusion.

In [30] an autonomous docking system for electric vehicles recharging is presented. It is based on an infrared camera installed in the infrastructure. A visual servoing system coupled with an automatic controller allows the vehicle to dock to the recharging booth in a street parking area. The prototype has shown good behavior in the city of Paris.

The paper in [31] proposes an approach for lane segmentation and tracking that is robust to varying shadows and occlusions. The results show that the proposed approach performs better than the traditional gradient-based approach under the difficulties caused by shadows and occlusions.

In [32] authors propose a technique that involves optical flow and driver's kinematics analysis to improve the robustness of the driver's alert state under pose changes using a single camera with NIR illumination. Author show the effectiveness of the approach in an experiment involving 15 persons in a simulator under different levels of sleep deprivation.

The ADAS proposed in [33] aims to prevent unsafe steering commands by means of a haptic handwheel. The paper addresses system requirements and provides implementation details to tele-operate two different off/on-axle combinations of a tracked mobile robot pulling and pushing two different trailers.
